# The Elements of the Dignity of Care Staff Who Work in Long Term Care Facilities

**DOI:** 10.1093/geroni/igaf122.2696

**Published:** 2025-12-31

**Authors:** Nanako Hasegawa, Cynthia Jacelon

**Affiliations:** Aichi Prefectural University, Nagoya, Aichi, Japan; University of Massachusetts Amherst, Amherst, Massachusetts, United States

## Abstract

There is limited research on the dignity of care staff, however, the dignity of care staff may be influenced by the work environment, the staff’s personal thoughts, and care experiences with patients. In turn, the care staff’s dignity is related to the delivery of dignified care. The inability to provide dignified care undermines the dignity of care staffs, and hurts their health. The purpose of this study was to clarify the elements of the individual dignity of care staff who work in long term care facilities. We integrated the findings of a systematic review focused on the individual dignity of staff with the findings of 18 hermeneutic phenomenological interviews of care staff in care at a long term care facility into a previously developed concept matrix of dignity. A total of 4242 titles were obtained from MEDLINE, Web of Science, and CINAHL using the keywords “Dignity,” “Respect,” and “Staff.” Based on predefined criteria, eighteen (n = 18) articles were selected for review. The concept matrix of dignity in care was used to classify the types of staff dignity according to their characteristics. The integrated findings of the care staff interviews, and systematic review indicated the dignity of care staff could be characterized by three themes: ‘The professional dignity,’ ‘Dignity in community,’ and ‘The individual dignity of the staff not affected by others.’ The specific elements of dignity for care staff represent the individual and their interactions with patients, other staff, the organization and society, providing clues for bringing dignified care into practice.

